# Prevalence and influencing factors of psychological distress among nurses in sichuan, china during the COVID-19 outbreak: A cross-sectional study

**DOI:** 10.3389/fpsyt.2022.854264

**Published:** 2022-08-04

**Authors:** Caixia Xie, Jia Zhang, Jia Ping, Xinyu Li, Yu Lv, Limei Liao

**Affiliations:** ^1^Department of Nursing, Sichuan Provincial People's Hospital, University of Electronic Science and Technology of China, Chengdu, China; ^2^Department of Neurosurgery, Sichuan Provincial People's Hospital, University of Electronic Science and Technology of China, Chengdu, China; ^3^General Practice Center, Sichuan Provincial People's Hospital, University of Electronic Science and Technology of China, Chengdu, China; ^4^Department of Healthcare-Associated Infections Control Center, Sichuan Provincial People's Hospital, University of Electronic Science and Technology of China, Chengdu, China; ^5^School of Medicine, University of Electronic Science and Technology of China, Chengdu, China; ^6^Chinese Academy of Sciences Sichuan Translational Medicine Research Hospital, Chengdu, China

**Keywords:** mental health, nurses, COVID-19, psychological distress, prevalence and influencing factors

## Abstract

**Background:**

The COVID-19 pandemic has spread across the world. Nurses have inevitably been influenced by it.

**Purpose:**

To investigate the prevalence and influencing factors of psychological distress among nurses in Sichuan, China over the COVID-19 outbreak.

**Methods:**

This study used a cross-sectional survey design. Thousand eight hundred and seventy nurses who worked in COVID-19-designated hospitals participated in the study during the pandemic. Data was collected online between February 8 and February 13, 2020. The self-designed General Information Questionnaire, the General Health Questionnaire-12, the Perception of Hospital Safety Climate Scale, and the Simplified Coping Style Questionnaire were used. The binomial logistic regression model was applied to assess the association between psychological distress and potential explanatory variables.

**Findings:**

At the beginning of the epidemy of the COVID-19 outbreak, 12% of nurses were found to experience psychological distress. The main influencing factors were personal precautionary measures at work, discomfort caused by protective equipment, perception of the hospital safety climate, coping style, and professional title.

**Conclusions:**

In the pandemic, wearing protective equipment correctly, a safe hospital climate, and positive coping style for nurses could be beneficial for nurses' mental health. Nurse managers should take measures to build a safe hospital climate.

## Introduction

The coronavirus (COVID-19) outbreak began in December 2019, resulting in significant loss of life across the world. Level 1 emergency status, the highest level, was announced, with the strictest infection control measures implemented. Sichuan, China, was affected by several cases from Wuhan and local transmission. Nurses were the primary implementers of the protective measures taken to control COVID-19 in Sichuan (1). In all three major coronavirus outbreaks of the last two decades (SARS, Ebola, and COVID-19), nurses' mental health has been affected (2). It was reported that during the COVID-19 outbreak, 34.4% of the medical and nursing staff working in Wuhan had mild psychological distress, 22.4% had moderate psychological distress, and 6.2% had severe psychological distress (3).

Psychological distress is an unpleasant emotional experience caused by several psychological (cognitive, behavioral, emotional), social, and spiritual factors. It can develop from and involve vulnerability, sadness, fear, anxiety, depression, social separation, and spiritual crisis (4). Psychological distress is reported to cause adverse effects on physical health including lowered immunity (5, 6), the inability to make the most accurate and optimal decisions for patients, which might impair their safety (7), reduced job and life satisfaction, and tension in interpersonal relationships (8, 9). Paying attention to the mental health of nurses during COVID-19 and exploring its influencing factors is essential for the formulation of mental health promotion strategies for nurses at both the individual and organizational levels. These will help nurses reduce any possible psychological distress and improve their mental health.

During the pandemic period of COVID-19, the psychological distress of nurses was affected by numerous individual and work-related factors, including their personality characteristics, age, gender, marital status, years of work experience, level of exposure to affected patients, self-efficacy, and presence of physical symptoms (2, 10, 11). However, the above studies did not explore whether characteristics of the workplace, such as the supply of protective materials, application of protective measures, and any possible discomfort caused by protective equipment, were influencing factors contributing to nurses' psychological distress.

Perception of a hospital's safety climate refers to employees' overall perception of the safety of their working environments (12). It was suggested that the perception of hospital climate may be related to nurses' psychological distress. Coping style refers to the method of dealing with stress and maintaining psychological balance. For nurses, participation in work related to COVID-19 is a significant stressor. It is suggested that nurses' different coping styles may have an impact on their psychological distress. Therefore, it is necessary to explore the influence of the supply of protective materials in the workplace, the application of personal protective measures, discomfort caused by protective equipment, perception of hospital safety climate, and coping style on nurses' psychological distress.

The main objectives of this study were to (1) describe the psychological distress of Chinese nurses in COVID-19-designated hospitals in Sichuan during the COVID-19 outbreak and (2) examine the main factors of psychological distress with a focus on work status, perception of hospital safety climate, and coping styles.

## Methods

### Design

This study used a cross-sectional survey design.

### Participants

The sample size of logistic regression (binary outcome) generally follows the principle of 10 events per variable. There were 13 independent variables to be included in this study, so the number of positive events was at least 14 × 10 = 130. As per past research, when SARS broke out, the incidence of psychological distress among nurses was 27.5% (13). Therefore, we used a positive event rate of 27.5% for the sample calculation. Considering the loss of 10–20% of the sample, the minimum sample size required for this study was *N* = 14 × 10 × (1+0.2)÷27.5% = 611.

From February 8 to February 13, 2020, during the COVID-19 outbreak in China, a convenient sampling method was used. One COVID-19-designated hospital each from five regions, East, South, West, North and middle, of the Sichuan Province were selected. Nurses from these five hospitals were invited to participate in the study, and 1,870 nurses volunteered.

### Data collection

Data was collected online through the Questionnaire Star platform, an online survey tool similar to Survey Monkey. Information about the investigation and the survey quick response code were sent through WeChat, a web-based social media application, to nurse managers in the five hospitals. This was then distributed to the nurses. The number of answers provided by the same IP address was limited, and each IP address could only answer the survey once. Therefore, repeat submissions and invalid data were effectively controlled. Four main questionnaires were used in this study. They are outlined as follows:

#### The general information questionnaire

The GIH is a self-designed instrument for demographic information and work status during the COVID-19 pandemic. The demographic characteristics included sex, age, marital status, number of children, nursing educational background, work year, and professional title. The work status information focused on direct contact with confirmed or suspected COVID-19 patients, the supply of protective materials in the workplace, application of personal protective measures, and any possible discomfort caused by protective equipment.

#### The general health questionnaire-12 (GHQ-12)

Psychological distress was measured using the 12-item GHQ-12, which is a widely used self-administered tool for emotional distress derived from the original 60-item version (14, 15). It consists of six positively phrased items and six negatively phrased items with four responses each, ranging from “better than usual” to “much less than usual.” A cut-off score of four was selected to identify the presence of psychological distress, defined as a break from normal functioning (e.g., loss of sleep, loss of self-confidence, or the inability to make decisions) (16). The reliability of the GHQ-12 in the general population ranged from 0.71 to 0.86 (17). The internal consistency of the GHQ-12 in this study was 0.85.

#### The perception of hospital safety climate scale (PHSCS)

The perception of hospital safety climate was measured using the revised Chinese version of the PHSCS (18), which was initially used in the context of organizational commitment to management projects to reduce blood-borne pathogen exposure risk (12). It consists of 21 items and five dimensions: management support with six items, obstacles to safe work with three items, feedback and training with six items, cleanliness and tidiness with three items, and conflict and communication with three items. Each item has a score ranging from 1 (strongly diagree) to 5 (strongly agree) as per a 5-point Likert scale. The lower the score, the better the perception of the hospital safety climate. With an assessment of 391 nurses conducted, the internal consistency and retest reliability of the revised Chinese version of PHSCS were reported to be 0.87 and 0.84, respectively (18). The internal consistency of the revised Chinese version of the PHSCS in this study was 0.84.

#### The simplified coping style questionnaire (SCSQ)

This questionnaire (19) was based on the Ways of Coping questionnaire (20). It is a 20-item self-report questionnaire that includes two dimensions: an positive coping style with 12 items and a negative coping style with eight items. The items measured typical coping attitudes and methods using a four-point Likert scale (0 = never; 1 = sometimes; 2 = often; 3 = always). The SCSQ has been commonly used in China, and its test-retest coefficient is 0.89. The internal consistency coefficients (Cronbach's alpha) were reported to be 0.89 and 0.78 for the active and positive coping dimensions (19). In this study, they were 0.929 and 0.830, respectively.

### Ethical considerations

This study was approved by the Human Research Ethics Committee of the Sichuan Provincial People's Hospital (Protocol No. 2020103). Completion of the online survey was considered consent to participate in the study, which was clearly stated in the instructions for the questionnaires.

### Data analysis

Analyses were performed using the SPSS 22.0 statistical program (IBM Corp., Armonk, NY, USA). Categorical variables were expressed with frequency and percentages, and continuous variables were expressed using mean and standard deviation (SD). A Pearson's chi-square test and independent Students' *t*-test were performed to identify potential explanatory variables for psychological distress. The binomial logistic regression model was applied to assess the association between psychological distress and potential explanatory variables while adjusting for other identified predictors. This was carried out using a sequential modeling approach. *P*-values <0.05 were considered to be statistically significant.

## Results

### Participant characteristics

Totally, 1,870 nurses participated in the study. Characteristics of the subjects are presented in Table 1.

**Table 1:** Characteristics of the subjects (*N* = 1,870).

**Variable**	**Frequency (*n*)**	**Proportion (%)**
**Gender**		
Male	69	3.7
Female	1,801	96.3
**Age (years)**		
<25	301	16.1
25~30	709	37.9
31~35	417	22.3
36~40	204	10.9
>40	239	12.8
**Marital status**		
Unmarried	664	35.5
Married	1,206	64.5
**Child/Children's situation**		
No	767	41
Yes	1,103	59
**Highest education**		
College and below	641	34.4
Undergraduate and above	1,229	65.6
**Work year**		
<5	499	26.7
5–10	730	39.0
11–15	276	14.8
16–20	120	6.4
>20	245	13.1
**Professional title**		
Registered nurse	498	26.6
Primary	868	46.4
Intermediate	419	22.4
Senior	85	4.5
**Protective supplies in your workplace**		
Sufficient	130	7.0
Basically sufficient	1,256	67.2
Not Sufficient	484	25.9
**Personal precautionary measures at work**		
Adequate	713	38.1
Basically adequate	1,026	54.9
Inadequate	131	7.0
**Discomfort caused by protective equipment**		
No discomfort	606	32.4
Somewhat discomfort	988	52.8
Discomfort	276	14.8
**Direct contact with confirmed or suspected cases**		
No	169	9.0
Possible	1,371	73.4
Yes	330	17.6

### Descriptive statistics

Table 2 displays the incidence of psychological distress, scores of perception of hospital safety climate and coping style in nurses. With scores of the GHQ-12 equal to or greater than 4, 225 nurses (12%) experienced psychological distress. The mean and SD of the total score of the PHSCS was 98.1 ± 10.5 with management support dimension of 28.2 ± 3.2; obstacles to safe work of 13.5 ± 2.2; feedback and training of 28.6 ± 2.9; cleanliness and tidiness of 13.6 ± 2.1; and conflict and communication of 14.2 ± 1.6. The mean and SD of the SCSQ with positive coping style was 24.7 ± 7.9; and the negative coping was 9.6 ± 5.3. The normality test showed that all quantitative data had normal distributions.

**Table 2:** Scores of the PHSCS and SCSQ, and percentages of psychological distress.

**Variables**	***N* (%)**	**Mean ±SD**	**Response range**
**Perception of hospital safety**		98.1 ± 10.5	
**Climate**			21–105
Management support		28.2 ± 3.2	6–30
Obstacles to safe work		13.5 ± 2.2	3–15
Feedback and training		28.6 ± 2.9	6–30
Cleanliness and tidiness		13.6 ± 2. 1	3–15
Conflict and communication		14.2 ± 1.6	3–15
**Positive coping**		24.7 ± 7.9	0–36
**Negative coping**		9.6 ± 5.3	0–24
**Psychological distress**		1.4 ± 1.7	0–2
No	1,646 (88.0%)		
Yes	224 (12.0%)		

### Univariate analysis

Pearson's chi-square tests and independent Student's *t*-tests were performed to identify potential variables for psychological distress. Table 3 compares the characteristics of the subjects between groups with and without psychological distress.

**Table 3:** Univariate analysis of psychological distress in nurses.

	**No. of respondents**				**Statistics**	
	**No psychological distress (*n*, %) (*n* = 1,645)**	**Psychological distress (*n*, %) (*n* = 225)**	**No psychological distress (Mean ±SD) (n = 1,645)**	**Psychological** **distress (Mean ±SD) (n = 225)**	***χ/t***	***P*-value**
**Gender**					2.593	0.107
Male	65 (4.0%)	4 (1.8%)				
Female	1,580 (96%)	221 (98.2%)				
**Age (years)**					38.454	<0.001
<25	286 (17.4%)	14 (6.2%)				
25~30	198 (12%)	41 (18.2%)				
31~35	642 (39%)	67 (29.8%)				
36~40	351 (21.4%)	66 (29.3%)				
>40	168 (10.2%)	37 (16.5%)				
**Marital status**					15.957	<0.001
Married	1,034 (62.9%)	172 (76.4%)				
Unmarried	611 (37.1%)	53 (23.6%)				
**Child/Children**					24.058	<0.001
no	709 (43.1%)	58 (25.8%)				
yes	936 (56.9%)	167 (74.2%)				
**Highest education**					5.131	0.024
College and below	579 (35.2%)	62 (27.6%)				
Undergraduate and above	1,066 (64.8%)	163 (72.4%)				
**Work year**					32.221	<0.001
<5	644 (39.1%)	86 (38.2%)				
5–10	468 (28.4%)	30 (13.3%)				
11–15	232 (14.1%)	45 (20%)				
16–20	98 (6%)	22 (9.8%)				
>20	203 (12.4%)	42 (18.7%)				
**Professional title**					34.071	<0.001
Registered nurse	460 (28%)	38 (16.9%)				
Primary	775 (47.1%)	94 (41.8%)				
Intermediate	347 (21%)	72 (32%)				
Senior	64 (3.9%)	21 (9.3%)				
**Protective supplies in your workplace**					35.984	<0.001
Sufficient	120 (7.3%)	10 (4.5%)				
Basically sufficient	1,136 (69.1%)	120 (53.3%)				
Not Sufficient	389 (23.6%)	95 (42.2%)				
**Personal precautionary measures at work**					60.909	<0.001
Adequate	670 (40.7%)	43 (19.1%)				
Basically adequate	881 (53.6%)	145 (64.4%)				
Inadequate	94 (5.7%)	37 (16.5%)				
**Discomfort caused by protective equipment**					54.827	<0.001
No discomfort	572 (34.8%)	34 (15.1%)				
Somewhat discomfort	861 (52.3%)	128 (56.9%)				
Discomfort	213 (12.9%)	63 (28%)				
**Direct contact with confirmed or suspected cases**						
No	1,241 (75.4%)	130 (57.8%)			31.566	<0.001
Possible	137 (8.3%)	32 (14.2%)				
Yes	267 (16.3%)	63 (28%)				
**Perception of hospital safety climate**			99.0 ± 10.0	91.6 ± 12.0	10.141	<0.001
**Positive coping**			25.1 ± 7.9	21.7 ± 6.6	6.252	<0.001
**Negative coping**			9.4 ± 5.4	11.0 ± 4.2	−4.222	<0.001

### Logistic regression analysis

Logistic regression analysis identified six factors that were significantly associated with the presence of psychological distress (see Table 4). Nurses without any professional title had 48.8% lower odds of developing psychological distress when compared with nurses with a senior professional title (OR 0.512, 95% CI 0.207–1.267). Inadequacy in personal precautionary measures at work resulted in a significantly increased risk of psychological distress (taking “adequate” as a reference, OR 1.753 for “basically adequate,” and OR 3.568 for “inadequate”). Discomfort caused by protective equipment was associated with an increased risk of psychological distress (taking taking “No discomfort” as a reference, OR 1.832 for “Somewhat discomfort,” and OR 3.137 for “Discomfort”). The higher the score of perception of hospital safety climate and positive coping, the lower the incidence of psychological distress. The higher the score of negative coping, the higher the incidence of psychological distress.

**Table 4:** Logistic regression analysis of psychological distress on nurses.

**Variable**	**B**	**Standard Error**	**Wald**	***P-*value**	**OR**	**95% CI**
**Professional title**						
Senior					1.000	
Intermediate	−0.774	0.348	4.963	0.026	0.461	0.233–0.911
Primary	−1.131	0.394	8.215	0.004	0.323	0.149–0.699
Registered nurse	−0.67	0.462	2.097	0.148	0.512	0.207–1.267
**Personal precautionary measures at work**						
Adequate					1.000	
Basically adequate	0.561	0.197	8.126	0.004	1.753	1.192–2.578
Inadequate	1.272	0.285	19.881	<0.001	3.568	2.040–6.242
**Discomfort caused by protective equipment**						
No discomfort					1.000	
Somewhat discomfort	0.605	0.213	8.041	0.005	1.832	1.206–2.784
Discomfort	1.143	0.246	21.519	<0.001	3.137	1.935–5.086
**Perception of hospital safety climate**	−0.032	0.006	29.437	<0.001	0.968	0.957–0.980
**Positive coping**	−0.073	0.011	41.519	<0.001	0.930	0.910–0.951
**Negative coping**	0.095	0.017	29.381	<0.001	1.099	1.062–1.138

## Discussion

### The prevalence of psychological distress

One interesting finding of the study was that 12% of the nurse respondents reported experiencing psychological distress, at the beginning of the epidemy of the COVID-19 outbreak. The study was conducted in COVID-19-designated hospitals in Sichuan, which was a region less affected by COVID-19. As of February 2020, it had recorded a total of 539 confirmed cases and three deaths. A recent study found that the prevalence of psychological distress among healthcare workers differed across regions with varying incidences of COVID-19 infections (21). This is reasonable because nurses in Sichuan may potentially feel safer than nurses in Hubei, for example, when evaluating the possibility of receiving a COVID-19 patient, since they are working in a less-affected area.

### Influencing factors of psychological distress

#### Personal precautionary measures at work

During COVID-19, taking personal precautionary measures at work was a crucial step for frontline nurses to avoid getting infected (22). The results revealed that the psychological distress of nurses with inadequate personal protective measures was 3.568 times higher than that of nurses with adequate personal protection. This suggests that the implementation of personal protective measures can predict nurses' psychological distress when dealing with such sudden infectious diseases. In this study, personal protective measures referred to the necessary preventive measures in different workplaces based on first-, second-, and third-level protection requirements, which play an important role in isolation protection and reducing the rate of nosocomial infection (23). For instance, the emergency department has to take the first level of protection, requiring nurses to wear work clothes, isolation clothes, work caps, disposable surgical masks, and latex gloves and carry out hand hygiene and standard prevention when caring for patients. In the fever and isolation clinics, nurses should wear medical protective masks, work clothes, protective clothing, work caps, and latex gloves and take droplet isolation and contact isolation based on the requirements of the second-level protection. When performing procedures that may produce aerosol in suspected or confirmed COVID-19 patients, nurses should be equipped with a face mask or comprehensive respirator on the basis of secondary protection, according to the requirements of third-level protection (24, 25). Due to the sudden nature of the outbreak, there was a lack of protection knowledge and skills (26), and thus nurses could not correctly apply protection measures at the beginning of the pandemic. For example, when wearing a protective mask, the air tightness did not meet the requirements needed (27), and when taking off protective clothing, exposure behavior often occurred (28). Therefore, it is particularly important to strengthen nurses' training in the correct implementation of protective measures (29).

#### Discomfort caused by protective equipment

The results of this study showed that more than half the nurses experienced some discomfort, and 14.8% of nurses felt constant discomfort, due to protective equipment. The psychological distress of nurses who felt discomfort caused by protective equipment was 3.14 times higher than that of nurses who did not feel it. Nurses must wear medical protective equipment to avoid catching COVID-19. This can cause several types of discomfort, such as (1) stuffiness and dyspnea, (2) decreased visual clarity and operation sensitivity, (3) insufficient diet and water intake at work, (4) facial pressure injury (30), and (5) a variety of skin problems such as acne, seborrheic dermatitis, and dry skin (31). In addition, the use of facial coverings also impairs direct communication and eye contact between nurses, their colleagues, and patients (32). Therefore, it is important to explore safe and effective strategies to reduce the discomfort and inconvenience caused by protective devices. It was proposed that the prophylactic use of thin hydrocolloid dressings on the bridge of the nose could effectively protect against pressure injuries when protective devices were used (33). Measures such as sweat absorption clothing and antiperspirant can be used to improve comfort and ease the burden faced by medical staff wearing protective clothing. Anti-fogging agents and indwelling films can work well to minimize goggle fogging (34). Research on the improvement of protective equipment, including protective masks and goggles, should be carried out in the future. It is also necessary to explore effective training and management strategies that will help reduce the discomfort caused by incorrect wearing of protective devices.

#### Perception of hospital safety climate

Perception of hospital safety climate refers to the employees' overall perception of the working environment, including safety decision making, safety practices, and safety procedures (12). In the 1990's, the “safety climate perception to nurse occupational safety management” (35) was first applied. The study reported that the perception of hospital safety climate directly affected the safety behavior of medical staff. The better the perception of hospital safety climate, the better the occupational protection behavior and the lower the occupational injury rate (36).

The regression analysis showed that the better the nurses' perception of a hospital's safety climate, the lower the incidence of psychological distress. During the pandemic period, nurses' perceptions of hospital safety climates were affected by many factors including the high risk of virus infection (37), sharp increase in the number of patients (38), prolonged working hours, lack of protective equipment, and safety promotion measures taken by hospitals (39). In the face of the pandemic, nurse managers should consider the importance of perception of hospital safety climate a priority for nurses' mental health, and they should take all recommended measures to improve it in a timely and effective manner. This can include training for greater protection knowledge and skills (40), establishment of an inspection system for protective devices before work, and provision of adequate protective equipment (39).

### Coping style

Positive coping refers to positive strategies to eliminate or avoid stressors or decrease stress (41), while negative coping refers to avoidance (e.g., ignoring problems) or deterioration rather than solving problems (42). The results of this study showed that 1,870 nurses had either positive (9.6 ± 5.3) and negative (1.4 ± 1.7) coping scores during the pandemic period, which indicated that the frontline nurses working in the hospital exhibited more positive responses.

Regression analysis of this study showed that positive coping was a protective factor for nurses' mental health, which is consistent with the results of a study by Ilić et al. (43). It may be that, in the pandemic, a sense of professional mission, professional honor (44), professional values (45), and self-esteem (46) helped nurses adopt a variety of positive coping styles (47). Of course, there were also some negative coping strategies demonstrated, such as fear or avoidance of patients with suspected or actual COVID-19 infections. Therefore, nursing managers should pay attention to the coping styles of nurses during such periods and guide them to adopt positive ones. Furthermore, negative coping styles can be reduced through training.

#### Professional titles

This study shows that the higher the professional title, the more severe the recorded mental health problems are. Those with higher professional titles have to demonstrate stronger critical care thinking abilities (3) and undertake more social roles, which leads to greater psychological pressure. For these reasons, they are more likely to experience psychological distress.

### Limitations

Our study has several limitations. First, the study used a cross-sectional design. A causal link between main influencing factors and psychological distress over the COVID-19 outbreak was not established in this study. Second, the data was collected over 5 days at the beginning of the epidemy, without any longitudinal follow-up. With the fluctuation of the pandemic situation, nurses' psychological distress could oscillate.

## Conclusions

At the beginning of the epidemy of the COVID-19 outbreak, the incidence of psychological distress was 12%. Personal precautionary measures at work, discomfort caused by protective equipment, perception of hospital safety climates, coping styles, and professional titles were the factors influencing nurses' psychological distress. When dealing with sudden infectious diseases such as COVID-19, nurse managers must ensure that the protective equipment provided is sufficient. They must also train nurses in the correct use of protective equipment while performing actual work. At the same time, medical institutions and nursing managers should take effective measures for safety decision making, safety practices, and safety procedures according to the current pandemic situation and the specific situations of medical institutions so as to improve nurses' perception of the hospital safety climate. Nurse managers should assess whether the mental state of nurses who usually use negative coping styles is suitable for COVID-19 work. The application of these measures may reduce the incidence of psychological distress among nurses during the COVID-19 outbreak. Any possible long-term psychological distress of nurses is worth further investigation.

## Data availability statement

The raw data supporting the conclusions of this article will be made available by the authors, without undue reservation.

## Author contributions

CX, JZ, and JP carried out the studies, participated in collecting data, drafted the manuscript and supervision whole process. LL, XL, and YL conducted data analysis and helped to draft the manuscript. All authors read and approved the final version of the manuscript.

## Conflict of interest

The authors declare that the research was conducted in the absence of any commercial or financial relationships that could be construed as a potential conflict of interest.

## Publisher's note

All claims expressed in this article are solely those of the authors and do not necessarily represent those of their affiliated organizations, or those of the publisher, the editors and the reviewers. Any product that may be evaluated in this article, or claim that may be made by its manufacturer, is not guaranteed or endorsed by the publisher.
